# AI-Guided Long Non-Coding RNA Target Discovery for Precision Medicine: Integrating GWAS, Multi-Omics, Experimental Validation, and RNA Therapeutics

**DOI:** 10.3390/biomedicines14081722

**Published:** 2026-07-31

**Authors:** Mia Yang Ang, Li Chen, Lanni Song, Leonard Lipovich, Siew Woh Choo

**Affiliations:** 1 Department of Biomedical Sciences, Jeffrey Cheah Sunway Medical School, Faculty of Medical and Life Sciences, Sunway University, Sunway City, Petaling Jaya 47500, Selangor, Malaysia; 2 Sunway Microbiome Centre, Faculty of Medical and Life Sciences, Sunway University, Sunway City, Petaling Jaya 47500, Selangor, Malaysia; 3 Zhejiang-Malaysia Joint Laboratory for Rare Medicinal Resources, Wenzhou-Kean University, 88 Daxue Road, Ouhai, Wenzhou 325060, China; 4 College of Science, Mathematics and Technology, Wenzhou-Kean University, 88 Daxue Road, Ouhai, Wenzhou 325060, China; 5 Dorothy and George Hennings College of Science, Mathematics and Technology, Kean University, 1000 Morris Ave., Union, NJ 07083, USA; 6 International Frontier Interdisciplinary Research Institute (IFIRI), Wenzhou-Kean University, 88 Daxue Road, Ouhai, Wenzhou 325060, China

**Keywords:** long non-coding RNA, lncRNA, artificial intelligence, machine learning, GWAS, multi-omics, RNA therapeutics, precision medicine, target discovery, population genetics, genomic epidemiology

## Abstract

**Background/Objectives:** Precision medicine requires translation of genetic and molecular variation into clinically actionable therapeutic targets. However, many disease-associated signals identified by genome-wide association studies (GWAS) reside in non-coding regulatory regions, making biological interpretation and therapeutic prioritization difficult. Long non-coding RNAs (lncRNAs) are regulatory molecules with growing relevance to disease mechanisms, biomarker discovery, patient stratification, and RNA-based therapeutics. This review presents a translational framework for AI-guided lncRNA target discovery, linking non-coding genetic signals to experimental validation and clinical implementation. **Methods:** This narrative review synthesizes literature on GWAS interpretation, quantitative trait loci analysis, epigenomic annotation, single-cell and spatial transcriptomics, multi-omics integration, artificial intelligence and machine learning, experimental validation, RNA therapeutic modality selection, delivery assessment, safety evaluation, and biomarker-informed precision medicine. **Results:** Genetic and multi-omics data can nominate disease-relevant lncRNAs, but no single evidence layer is sufficient to establish causality, mechanism, druggability, or clinical utility. AI can integrate heterogeneous biomedical datasets, rank candidate lncRNAs, detect regulatory patterns, and prioritize transcripts for validation. However, computational prediction should be interpreted as decision support rather than proof of therapeutic relevance. Candidate targets require disease-context expression validation, functional perturbation, mechanistic assessment, appropriate model systems, therapeutic modulation, delivery-feasibility assessment, safety evaluation, and patient-selection strategies. **Conclusions:** LncRNAs represent a promising but challenging therapeutic target class. A responsible translational pipeline should connect non-coding genetic evidence and multi-omics support with AI-guided prioritization, experimental validation, RNA therapeutic strategy selection, delivery assessment, safety evaluation, and clinical implementation. The framework defines qualification criteria and decision gates to reduce premature target claims during translational development.

## 1. Introduction

Genome-wide association studies, population sequencing, and multi-omics profiling have dramatically expanded the catalogue of disease-associated loci. However, over 98% of the 3.3 GB of DNA that comprises the human genome sequence is outside of protein-coding gene exons [1], and hence, more than 95% of significantly disease-associated human genetic variants are non-coding [2]. A necessary consequence of this conundrum is that many signals remain difficult to translate into therapeutic targets because they occur in non-coding regulatory regions that do not easily lend themselves to obvious functional interpretation [3]. Long non-coding RNAs (lncRNAs) are central to this challenge because they can regulate chromatin state, transcription, RNA stability, molecular interactions, and disease-associated cellular phenotypes, often in a tissue- or context-specific manner [4]. However, they have traditionally been overlooked in protein-centric GWAS annotations. Most importantly, their relevance is underscored by their sheer abundance: among currently catalogued human gene annotations, lncRNA genes represent a large majority of non-protein-coding gene models [5]. However, a lncRNA transcribed from a disease-associated genomic interval is not automatically a therapeutic target; its translational relevance depends on causal evidence, disease-context expression, functional perturbation outcomes, and disease-relevant mechanisms, and even when those criteria are met, target accessibility, delivery feasibility, and clinical safety will determine the therapeutic utility, if any, of that lncRNA as a drug target [6]. Despite the large number of annotated human lncRNA genes, currently approved oligonucleotide therapeutics predominantly target protein-coding transcripts, splice events, or small RNAs rather than lncRNAs. This review addresses this critical gap by examining how artificial intelligence (AI)-guided prioritization can be integrated with biological validation and RNA therapeutic development to support more credible lncRNA target discovery for precision medicine.

Prior reviews have primarily examined lncRNA biology and disease mechanisms [4,7,8], computational prediction of lncRNA–disease associations [9,10,11], post-GWAS causal gene prioritization using explainable AI [12], or ncRNA and lncRNA therapeutic development [6,13,14]. The distinctive contribution of this review is to connect these domains through an evidence-aware translational framework that distinguishes association from causal and therapeutic support, defines the experimental and mechanistic qualification required for AI-prioritized candidates, and introduces explicit decision gates for therapeutic modality compatibility, delivery feasibility, safety, and patient stratification before candidate advancement.

### Rationale for an AI-Guided Translational Framework

**Figure 1:**
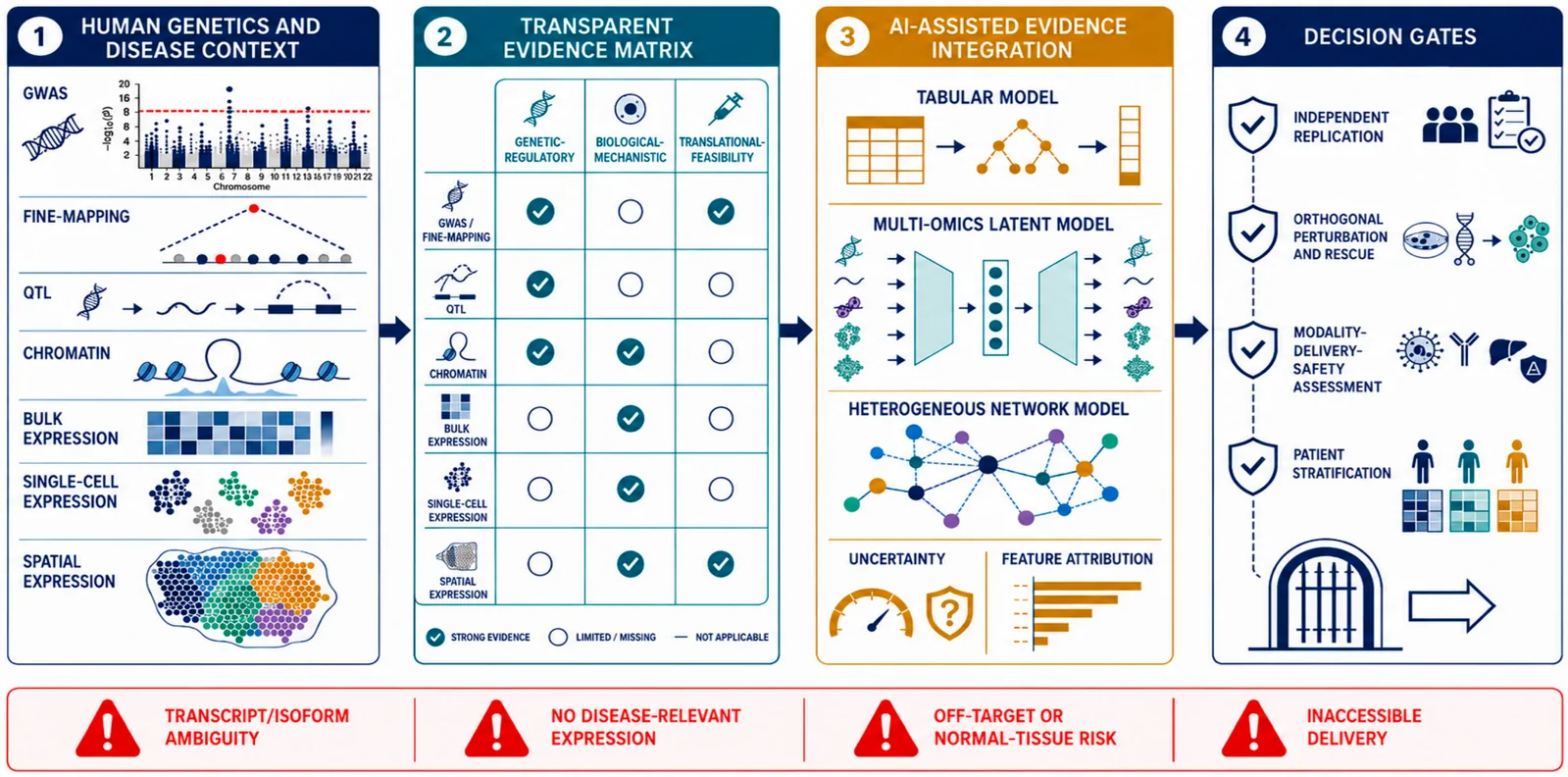
Translational workflow for AI-guided lncRNA therapeutic target discovery. Disease-associated non-coding variants identified through GWAS, fine-mapping, quantitative trait locus analysis, and regulatory annotation are linked to candidate lncRNAs and evaluated using transcriptomic, epigenomic, single-cell, spatial, interaction, and clinical evidence. AI and machine-learning approaches integrate these evidence layers to rank candidates, estimate uncertainty, identify the principal supporting features, and highlight evidence gaps. Prioritized lncRNAs then undergo sequential qualification through transcript and isoform confirmation, disease-context expression analysis, orthogonal functional perturbation, mechanistic validation, therapeutic modality and delivery assessment, target-engagement measurement, safety evaluation, and patient-stratification planning. Candidates should be downgraded when transcript identity is uncertain, disease-relevant expression is absent, RNA-mediated effects cannot be distinguished from locus-mediated effects, validation is dependent on a single perturbation method, or no feasible delivery and safety strategy is available. AI-guided prioritization therefore supports, but does not replace, biological and translational validation.

The central challenge addressed in this review is how non-coding genetic and molecular signals can be converted into credible and testable lncRNA therapeutic hypotheses. GWAS and multi-omics studies can discover and prioritize disease-associated loci and putative candidate transcripts, but association alone is insufficient to establish causal function, therapeutic relevance, or clinical utility [15]. AI and machine learning can support candidate prioritization by integrating genetic, transcriptomic, epigenomic, single-cell, spatial, interaction, and clinical data. However, computational ranking serves solely as decision support, rather than proof of causality or druggability. Candidate lncRNAs therefore require a concise, stepwise qualification process before therapeutic claims are made. By connecting GWAS and multi-omics evidence with experimental validation, RNA therapeutic strategy selection, and clinical implementation, this approach aims to reduce premature target claims while preserving the value of scalable computational evidence synthesis. The proposed translational workflow is summarized in Figure 1, which illustrates how disease-associated non-coding genetic signals located within or near lncRNA loci, or acting through their regulatory elements, can be progressively evaluated and refined into candidate lncRNA therapeutic targets.

## 2. Long Non-Coding RNAs as Disease Regulators and Therapeutic Candidates

LncRNAs are increasingly recognized as disease-relevant regulatory molecules with clear value in biomarker discovery, patient stratification, and RNA-based therapeutic development. They are commonly defined as transcripts longer than 200 nucleotides with limited conventional protein-coding capacity, although current annotations recognize substantial isoform diversity, transcript heterogeneity, and small open reading frames [16,17]. This complexity is important for therapeutic target discovery because the biological and pharmacological interpretation of a candidate lncRNA depends on the exact transcript isoform(s) involved, genomic locus and strandedness, regulatory relationships with nearby or overlapping genes, such as sense–antisense pair formation and bidirectional promoter sharing, expression context, subcellular localization, and molecular mechanism [18].

In translational biomedicine, lncRNAs should be considered as neither automatically functional nor automatically druggable [8]. Some lncRNAs regulate chromatin organization, transcription, RNA stability, protein interactions, signaling pathways, and disease-associated cellular phenotypes, whereas others may reflect enhancer activity (enhancer RNAs, eRNAs, transcribed from promoters in enhancers to regulate the activity of distant protein-coding genes), or be mere transcriptional noise or outcomes of secondary changes in disease tissue. Therefore, the central question is not only whether a lncRNA is associated with disease, but whether it causally contributes to disease biology in a way that can be measured, perturbed, therapeutically modulated, and translated into patient benefit.

### 2.1. Classification, Annotation, and Target-Relevant Features of lncRNAs

LncRNAs can be classified into biotypes according to genomic position, transcriptional orientation relative to nearby or overlapping genes, and, where known, molecular mechanism or regulatory function. Major positional classes include long intergenic non-coding RNAs (lincRNAs, transcribed from “gene deserts” far from other genes), antisense transcripts that overlap coding or non-coding genes on the opposite genomic DNA strand, intronic lncRNAs, bidirectional promoter-associated lncRNAs, enhancer-associated transcripts, including eRNAs, and pseudogene-derived lncRNAs. These categories are useful for therapeutic target discovery because they help define whether a candidate lncRNA may act independently, regulate a nearby or overlapping protein-coding gene, reflect enhancer activity directed toward a distant protein-coding target, or, if transcribed from a pseudogene, have a function that relates to or affects the pseudogene’s parental gene.

Current annotation resources are essential for avoiding imprecise target claims. GENCODE [5] and NONCODE [19] provide a reference human gene catalog incorporating lncRNA genes and updated lncRNA annotation resources. LncBook [20] and LNCipedia [21] support transcript-level lncRNA annotation. RNAcentral [22] integrates non-coding RNA sequences, and lncATLAS [23] supports subcellular localization assessment. These resources are not merely descriptive databases; they inform experimental design by defining and cataloguing transcript boundaries, isoforms, genomic overlaps, and subcellular localization. For example, a nuclear lncRNA may be more compatible with RNase H-dependent antisense oligonucleotides or transcriptional modulation, whereas a cytoplasmic lncRNA may be more suitable for RNA interference-based strategies [14].

Several biological features make lncRNAs attractive for precision medicine. These include highly tissue-restricted expression, exquisite cell-type specificity, disease-associated regulation, regulatory versatility, and potential detectability as biomarkers. Furthermore, the primate-specific evolutionary origin of the vast majority of human lncRNA genes [24,25], together with the basic finding that poor lncRNA sequence conservation does not imply a lack of function [26], indicates that lncRNAs may offer advantages over more highly conserved mRNAs and protein targets. Specifically, sequence-based drugs targeting these lncRNAs may be less likely to produce off-target effects arising from unintended interactions with ancient, conserved genes. However, some of these same features also create translational challenges [27]. Low abundance, isoform ambiguity, poor conservation, overlapping transcription, and species-specific function can complicate assay design, model selection, and interpretation of perturbation experiments. Therefore, therapeutic investigations should specify the relevant lncRNA transcript ID or isoform, the genome assembly used in the study, the strand or direction of transcription, and the disease-relevant cellular context whenever possible. The major lncRNA classes most relevant to therapeutic target discovery are summarized in Table 1, with emphasis on their defining features, translational relevance, limitations, and representative references.

### 2.2. Mechanisms of lncRNA-Mediated Disease Regulation

LncRNAs influence disease biology through diverse mechanisms, including chromatin remodeling, enhancer–promoter communication, transcriptional regulation, regulation of RNA processing, mRNA stability, and translation, protein scaffolding, and RNA-RNA interactions. Nuclear lncRNAs may recruit chromatin regulators, shape local regulatory architecture, influence R-loop formation, or alter topologically associated domain organization. Polycomb-associated lncRNAs remain important examples in cancer biology, but recruitment models require direct evidence of RNA–protein binding, chromatin change, and downstream phenotypic effects.

Cytoplasmic lncRNAs may regulate transcript stability, translation, signaling complexes, or competing endogenous RNA (ceRNA) networks. However, predicted microRNA binding or pathway association alone is insufficient to establish a mechanism. CeRNA models, in particular, require quantitative validation under endogenous expression conditions because computational interaction predictions can overstate biological relevance. RNA structure is also increasingly important, as RNA folding can influence protein binding, antisense oligonucleotide accessibility, and small-molecule recognition [33]. Nevertheless, beyond well-characterized classical RNAs such as rRNAs and tRNAs, structural characterization of the lncRNAome and RNA–protein complexes still lags far behind advances in protein complex structure prediction, even in the current post-AlphaFold era.

Over 20 years ago, Yoshihide Hayashizaki, who led the FANTOM Consortium [34] effort to build complete empirically defined human and mouse lncRNA catalogs, remarked that, for some lncRNAs, their presence, that is, their transcription, may matter more than their sequence [35]. A key translational issue is that different perturbation strategies may test different biological mechanisms, some, but not all, of which may have disease relevance in each context. Because of this, deleting a lncRNA locus, blocking its promoter, degrading the mature RNA, editing an enhancer, or inhibiting an RNA–protein interaction may produce different outcomes. Therefore, a target claim should specify whether the therapeutically relevant entity is the mature RNA transcript, the act of transcription, the DNA regulatory element, a structural RNA motif, or an associated molecular complex. This distinction is essential for matching biological mechanism to therapeutic modality.

### 2.3. lncRNAs as Biomarkers, Disease Drivers, and Therapeutic Targets

While lncRNAs can serve distinct biomedical roles, these roles should not be conflated [36]. A lncRNA may be useful as a biomarker if its expression reflects disease state, tissue injury, prognosis, molecular subtype, or treatment response. However, in the RNA field, yesterday’s biomarkers can become today’s drug targets once their mechanisms are fully elucidated [37]. Nevertheless, biomarker value does not automatically imply causal function or therapeutic targetability. Analytical validation, independent clinical cohorts, disease specificity, and clinical utility are required before biomarker claims can support precision medicine applications.

Numerous lncRNAs have been investigated as disease drivers or therapeutic candidates. These include HOTAIR in cancer metastasis [38], MALAT1 [39] and NEAT1 [40] in tumorigenesis, H19 in pulmonary fibrosis [41], ANRIL in atherosclerosis [42], GAS5 as a tumor-suppressive lncRNA in cancer [43], PVT1 as a metastasis- and prognosis-associated lncRNA [44], and LINC00607 in endothelial angiogenic function [45]. These examples demonstrate the biomedical relevance of lncRNAs, but they also illustrate the need for disease-specific validation. The fact that a specific lncRNA is widely studied is not, by itself, evidence of therapeutic relevance. NEAT1 and MALAT1 are among the highly expressed human lncRNAs, with broad expression across many cell types; this may partly explain the large number of studies focusing on these two lncRNAs. A candidate lncRNA must show reproducible disease-context expression, functional perturbation effects, mechanistic plausibility, and feasible therapeutic modulation.

For this reason, lncRNAs should be advanced as therapeutic targets only when perturbation changes a disease-relevant phenotype in an interpretable direction and in an appropriate biological model [13]. Candidates with strong expression association but limited functional evidence may still be valuable as biomarkers or disease-state reporters. By contrast, candidates supported by reproducible disease-context expression, mechanistic evidence, target accessibility, and feasible therapeutic modulation provide a stronger basis for RNA therapeutic development. Distinguishing diagnostic association from causal function and therapeutic targetability is therefore essential for responsible translation of lncRNA insights into post-genomic drugs.

## 3. From GWAS Signals to Candidate lncRNA Therapeutic Targets

GWAS can identify statistically significant disease-associated loci for common and less common diseases across and within population cohorts, highlighting genetic variants, typically single nucleotide polymorphisms (SNPs), in which specific alleles are associated with the disease or trait under study [46]. However, translating a non-coding association signal into a candidate lncRNA therapeutic target requires several additional steps. The key question is whether the same variant, credible set, or regulatory element plausibly links disease risk to lncRNA expression, splicing, chromatin state, cellular phenotype, or therapeutic response. This is particularly important because non-coding variants may act through distal enhancers, promoter regulation, chromatin architecture, RNA processing, or cell-type-specific regulatory circuits, rather than through the nearest annotated protein-coding gene.

For lncRNA target discovery, GWAS evidence should therefore be interpreted as an entry point rather than as direct evidence of therapeutic relevance. This caution also applies to protein-coding loci. For example, *FTO*, which harbors obesity-associated genetic variants, was initially considered a causal candidate for high BMI and obesity. However, subsequent studies showed that the association resulted from intragenic non-coding variants within *FTO* that pointed to an enhancer regulating another, more distant protein-coding gene, *IRX3*, which was ultimately implicated in the phenotypes associated with genetic risk at that locus [47]. A candidate transcript becomes more compelling when genetic association converges with regulatory annotation, QTL evidence, disease-context expression, readiness for perturbation, and plausible therapeutic modulation [48]. Without this convergence, a lncRNA located near a disease-associated variant may represent a correlated transcript or a marker of local regulatory activity rather than a causal disease target.

### 3.1. Interpreting Non-Coding Association Signals

Many disease-associated variants map to regulatory regions, and functional annotation is needed because a variant can influence disease biology without altering protein sequence. Nearest-coding-gene assignment, traditionally a mainstay of GWAS interpretation, is especially limited for lncRNA discovery because the relevant transcript may be distal, antisense, enhancer-associated, cell-type-specific, or embedded within a broader regulatory domain. Regional visualization tools such as LocusZoom [49] can help inspect association patterns, but visualization alone does not establish causal variants, causal transcripts, or therapeutic mechanisms.

Fine-mapping and functional annotation improve interpretation by narrowing credible variant sets and identifying regulatory features that may influence disease-relevant transcription [50]. Tools such as FINEMAP [51] can prioritize plausible causal variants from GWAS summary statistics, while FUMA [52] can integrate positional, eQTL, chromatin, and functional annotations for candidate locus interpretation. These tools are useful for organizing evidence, but their outputs still require biological interpretation, tissue-relevance assessment, and experimental follow-up.

Population and tissue context are also important. Linkage disequilibrium patterns, allele frequencies, regulatory effects, and expression architecture can differ across ancestry groups, while regulation and expression of the same transcriptional unit can differ among tissues and even between adjacent cells within the same tissue. Therefore, candidate lncRNA prioritization should account for ancestry diversity and genetic admixture, as well as disease-relevant cell types and tissue-specific regulatory maps. This is essential for precision medicine because a regulatory signal discovered in one population or tissue may not transfer directly to another clinical context.

### 3.2. Mapping Risk Loci to lncRNA Transcripts and Regulatory Elements

Mapping disease-associated loci to lncRNAs should begin with accurate transcript annotation. Candidate assessment should record the precise localization of significant disease-associated genetic variants from GWAS with respect to the lncRNA locus, including whether they are outside or within the locus, exonic, intronic, splice-site-associated, or regulatory. It should also record the genome assembly version, lncRNA gene symbol, preferably from RefSeq or GENCODE, the corresponding gene identifier, such as an ENSG identifier, transcript identifier, such as an NR_ transcript accession from RefSeq or an ENST transcript identifier from GENCODE, strand, overlap with protein-coding genes if present, promoter location, proximity to known enhancers, and disease-relevant expression context. This is important because lncRNA annotation continues to evolve with long-read third-generation sequencing and updated reference resources [30].

A stronger mapping strategy combines several evidence layers. First, credible variants may reside directly within lncRNA promoters, exons, splice sites, enhancers, regions marked by DNA methylation or histone modifications, and/or chromatin interaction domains. Second, QTL evidence can test whether disease-associated variants are linked to lncRNA expression, splicing, chromatin accessibility, or methylation in relevant tissues [53]. Third, colocalization can assess whether the same genetic signal is likely to explain both the disease association and molecular regulation [54].

Cell-type resolution is increasingly important because lncRNA expression may be restricted to specific cell subpopulations. Bulk-tissue data can obscure disease-relevant signals if the candidate transcript is expressed only in immune cells, endothelial cells, stromal cells, neurons, adipocytes, or tumor subclones. Single-cell and single-nucleus datasets can therefore strengthen candidate mapping by showing whether the lncRNA is expressed in the relevant cells within the tissue and in the cellular compartment where disease biology occurs.

### 3.3. Prioritizing GWAS-Derived lncRNA Candidates for Therapeutic Validation

Candidate lncRNAs should be prioritized according to the strength and convergence of evidence rather than by genomic proximity alone [55]. A transcript supported only by location near a GWAS signal should rank below one supported by fine-mapping, colocalized eQTL evidence, disease-relevant expression, regulatory annotation, and functional plausibility [56]. A high-priority candidate should have a defined disease context, a plausible direction of effect, an experimentally testable mechanism, and a feasible therapeutic modulation strategy [57]. Future work should develop disease-specific, empirically calibrated prioritization approaches while preserving non-compensatory biological and translational decision gates. Explainable post-GWAS approaches can further organize evidence into inspectable variant-to-transcript, transcript-to-pathway, and pathway-to-phenotype chains while making uncertainty explicit and preserving the requirement for experimental validation [12].

The prioritization process can be organized into three overarching tiers—genetic–regulatory, biological–mechanistic, and translational—which are operationalized through five evidence domains: genetic evidence, regulatory evidence, disease-context evidence, functional and mechanistic evidence, and therapeutic feasibility. The genetic–regulatory tier includes association strength across populations, credible-set refinement, fine-mapping, colocalization, chromatin annotation, and tissue-relevant QTL evidence. The biological–mechanistic tier includes disease-context expression, cell-type specificity, pathway coherence, functional perturbation, and mechanistic validation. The translational tier includes target accessibility, therapeutic-modality compatibility, delivery feasibility, safety considerations, pharmacodynamic assessment, and biomarker potential. A candidate may remain valuable as a biomarker even when it does not yet satisfy the requirements for therapeutic advancement.

Examples such as the antisense lncRNA CDKN2B-AS1 illustrate why multi-layered evidence is needed [58]. A SNP–lncRNA association becomes more persuasive when genetic, regulatory, expression, and mechanistic data converge on the same disease-relevant pathway rather than merely the same genomic region. The goal is therefore not to generate the longest possible list of lncRNA candidates, but to identify a smaller set of transcripts with sufficient evidence to justify functional testing and therapeutic feasibility assessment. To facilitate computational workflow design for systematic candidate selection, Table 2 outlines a simplified evidence framework for prioritizing GWAS-derived lncRNAs according to genetic, regulatory, disease-context, functional, and therapeutic-feasibility evidence.

### 3.4. Operationalization and Relationship to Gene–Disease Curation Frameworks

These three tiers and five evidence domains can be operationalized as a disease-specific decision framework rather than as a universal numerical score. Because data availability, genetic architecture, and therapeutic constraints vary across diseases, fixed weights may imply that strong evidence in one domain can compensate for a critical weakness in another. Instead, genetic and regulatory support, disease-context relevance, functional and mechanistic evidence, and translational feasibility should be assessed separately using non-compensatory decision gates [63].

AI models may integrate available features, rank candidates, estimate uncertainty, and identify supporting or missing evidence [64,65]. However, a high computational score should not override uncertain transcript identity, absent disease-relevant expression, inadequate orthogonal perturbation and rescue, or the lack of a feasible therapeutic modality or delivery strategy. This is particularly important for lncRNAs because transcript knockdown, promoter repression, and genomic-locus deletion may test different biological entities [66].

The framework is informed by ClinGen gene–disease curation, which evaluates structured genetic and experimental evidence, but extends it for lncRNA therapeutic prioritization [63]. Additional requirements include transcript- and isoform-level certainty, distinction between RNA-mediated and locus-mediated effects, disease-relevant cellular expression, model concordance, modality compatibility, delivery feasibility, target engagement, and preliminary safety assessment [13,14,30]. Candidates supported only by genomic proximity, bulk-tissue expression, or a single perturbation method should therefore be retained for further validation rather than advanced as therapeutic targets.

### 3.5. Illustrative Case Study: CDKN2B-AS1/ANRIL at the 9p21 Locus

CDKN2B-AS1, also known as ANRIL, illustrates why strong human genetic evidence does not automatically establish a lncRNA as a therapeutic target. The chromosome 9p21 locus is reproducibly associated with atherosclerotic cardiovascular disease, and ANRIL expression has been linked to risk at this locus [58]. However, these findings do not identify the therapeutically relevant transcript, establish the direction of effect, or demonstrate that the mature RNA mediates the association.

Interpretation is complicated by multiple ANRIL isoforms, antisense transcription, and possible effects on neighbouring genes and regulatory elements [42]. Progression would therefore require isoform-resolved expression and regulatory analysis in relevant vascular or immune-cell states, together with complementary RNA- and locus-directed perturbations, rescue experiments, and mechanism-linked phenotypic readouts.

ANRIL also lacks sufficient evidence of feasible delivery, target engagement, therapeutic benefit, and safety in relevant vascular tissues. Under the proposed framework, it should therefore be regarded as a genetically supported candidate requiring further mechanistic and translational qualification rather than as an established therapeutic target.

Additional examples illustrate how lncRNA candidates may reach different stages of translational qualification. LINC03045 was identified through CRISPR interference screening and functionally linked to glioblastoma invasion, providing disease-relevant functional evidence but not yet complete therapeutic qualification [61]. In another example, systemic antisense targeting of NIS-lncRNA produced preclinical effects in a neuropathic-pain model, demonstrating therapeutic-modality selection and in vivo target modulation, although this candidate did not emerge from a complete GWAS-to-AI discovery pathway [67]. Together with ANRIL, these examples demonstrate that human genetic prioritization, functional validation, and therapeutic feasibility represent distinct stages of candidate qualification and should not be conflated.

## 4. Multi-Omics Integration for lncRNA Target Discovery

Multi-omics integration is increasingly used to connect genomic alterations with transcriptomic, proteomic, metabolic, spatial, and clinical phenotypes, although cross-platform heterogeneity and limited external validation remain important translational barriers [68]. For lncRNA target discovery, such integration is essential because lncRNA function cannot be inferred from genomic location or expression change alone. A candidate lncRNA may be linked to disease through genetic regulation, chromatin state, transcript abundance, RNA structure, molecular interactions, cell-type specificity, spatial localization, or clinical phenotype. For therapeutic prioritization, these layers should be interpreted together to determine whether independent evidence supports the same disease-relevant mechanism.

A useful multi-omics framework should therefore distinguish supportive association from translational evidence. Transcriptomic and epigenomic data can identify disease-context activity; proteogenomic, translatomic, and interaction data can clarify molecular function; single-cell and spatial technologies can define the relevant cellular niche; and clinical phenotyping can connect candidate lncRNAs to disease severity, prognosis, treatment response, or patient stratification. The strongest candidates are those for which these data layers converge on a plausible mechanism that can be tested experimentally and linked to therapeutic feasibility.

### 4.1. Public Data Resources and Data Harmonization

AI-guided lncRNA target discovery depends on the integration of public resources that provide complementary information on transcript annotation, sequence, subcellular localization, genetic regulation, chromatin state, disease-associated expression, clinical phenotype, and functional validation. However, these resources differ in annotation release, genome build, transcript resolution, tissue context, sample composition, and data-processing procedures. Their outputs should therefore be harmonized before model development or candidate prioritization, with explicit documentation of transcript identifiers, genomic coordinates, annotation versions, and disease-relevant cellular context. Table 3 summarizes representative public resources, their primary applications, lncRNA-specific limitations, and recommended safeguards.

### 4.2. Transcriptomic and Epigenomic Evidence

Transcriptomic data provide the first layer of evidence for determining whether a candidate lncRNA is expressed in disease-relevant tissues, cell types, or molecular subgroups. Bulk RNA-seq can identify disease-associated expression patterns, but it may obscure cell composition and isoform-specific regulation, particularly when pathogenic cell populations are rare or spatially restricted. Long-read transcriptomics can improve lncRNA interpretation by resolving transcript boundaries, splice isoforms, overlapping genes, and annotation uncertainty, all of which are important for designing targeted perturbation assays [72].

Epigenomic data add regulatory context by identifying active enhancers, promoters, accessible chromatin regions, DNA methylation changes, and histone-mark profiles at or near lncRNA loci [70]. These data help determine whether a candidate lncRNA is embedded within a disease-relevant regulatory program rather than being an isolated expression signal. For example, enhancer–gene regulatory maps can reveal disease mechanisms that are not captured by protein-coding annotation alone.

Transcriptomic and epigenomic evidence is strongest when expression, chromatin state, variant regulation, and disease phenotype point to the same biological context. If a lncRNA is differentially expressed but lacks regulatory support, it may still be useful as a biomarker but should not be treated as a therapeutic target without further functional evidence. Conversely, when significantly disease-associated variants, chromatin accessibility, enhancer activity, and disease-phenotype-relevant transcript expression converge on the same lncRNA locus, the candidate becomes more suitable for experimental validation.

### 4.3. Proteogenomic, Translatomic, and Molecular Interaction Evidence

Proteogenomic and translatomic evidence is important because some transcripts annotated as lncRNAs may contain small open reading frames or produce functional micropeptides [73], which have been directly validated by mass spectrometry [16]. Bifunctional lncRNAs are not new, as SRA1, the steroid receptor co-activator, has long been known to encode functionally distinct coding and non-coding transcript isoforms, a scenario that has subsequently been observed for other genes [74]. This possibility affects therapeutic interpretation because the relevant target may be the RNA transcript, the peptide product of its small ORF, or a regulatory locus linked to both RNA and protein output. Therefore, candidate evaluation should clarify whether therapeutic modulation is intended to alter mature RNA abundance, transcriptional activity, RNA structure, peptide production, or a broader regulatory complex.

Molecular interaction evidence can further define lncRNA mechanism [75]. RNA–protein and RNA–RNA interaction analyses, chromatin interaction profiling, and RNA structure probing using techniques such as PARS [76] can identify the molecular partners and structural features that support disease-relevant function. However, interaction alone is not sufficient to prove mechanism. A convincing target claim requires evidence that the interaction affects a disease-relevant pathway or phenotype.

These data are also directly relevant to therapeutic design [77]. ASO binding, siRNA design, CRISPR guide selection, and small-molecule targeting depend on transcript accessibility, subcellular localization, RNA structure, and molecular complex formation. Therefore, proteogenomic, translatomic, and interaction evidence should not be treated only as mechanistic annotation; they also help determine whether a candidate lncRNA can be modulated using a realistic therapeutic modality. Experimental validation of disease-candidate lncRNAs in a breast cancer reverse-genetics screen highlighted practical limitations in siRNA-mediated depletion of some cytoplasmic lncRNA targets [78].

### 4.4. Single-Cell, Spatial, and Clinical Multi-Omics Integration

Single-cell and spatial omics are particularly valuable for lncRNA target discovery because many lncRNAs show cell-type-specific or state-specific expression [79]. Bulk-tissue analysis may miss a candidate transcript if it is restricted to specific cell types, such as immune cells, endothelial cells, stromal compartments, neurons, or tumor subclones, or to disease-associated cell states. Single-cell transcriptomics can identify the specific cellular population in which the lncRNA is active, while spatial transcriptomics can determine whether expression occurs in the anatomical niche where pathology develops.

Clinical integration is required before a candidate lncRNA can be considered translationally meaningful. Candidate transcripts should be assessed against disease severity, molecular subtype, prognosis, therapeutic resistance, treatment response, or patient-selection markers. Cancer data portals and disease cohort datasets can support hypothesis generation, but clinical association still requires independent validation and appropriate adjustment for confounding variables.

**Figure 2:**
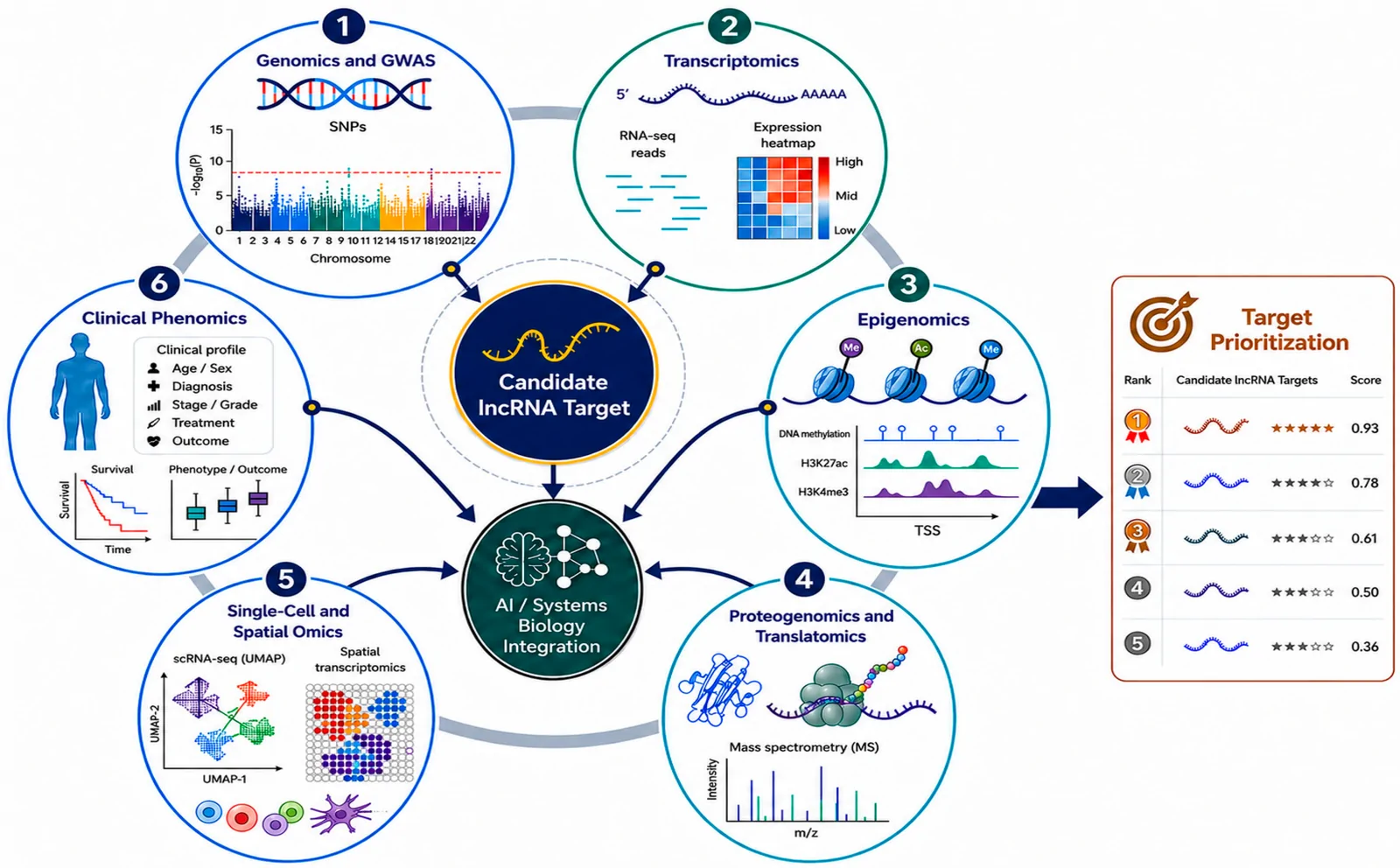
Multi-omics evidence layers for prioritizing candidate lncRNA therapeutic targets. Genetic, transcriptomic, epigenomic, proteogenomic, translatomic, single-cell, spatial, and clinical data provide complementary evidence for candidate prioritization. AI and systems–biology approaches can integrate these layers to identify transcripts for experimental validation, therapeutic feasibility assessment, and precision medicine development.

In this context, AI and systems–biology approaches can help integrate genetic, transcriptomic, epigenomic, proteogenomic, single-cell, spatial, and clinical data into a ranked evidence profile. A clinically useful candidate should show not only multi-omics association, but also disease-context expression, mechanistic plausibility, therapeutic accessibility, and measurable relevance to patient stratification or treatment response. Figure 2 summarizes how genetic, transcriptomic, epigenomic, proteogenomic, translatomic, single-cell, spatial, and clinical data layers can be integrated to support candidate lncRNA prioritization and downstream validation.

## 5. AI and Machine Learning for lncRNA Target Prioritization

Artificial intelligence (AI) and machine learning (ML) can support lncRNA target discovery by integrating heterogeneous biomedical evidence and ranking candidate transcripts for experimental validation [64]. Their value lies in prioritization, pattern recognition, and evidence synthesis rather than autonomous target discovery. This distinction is important because an AI-ranked lncRNA may be statistically plausible but still lack causal function, therapeutic accessibility, or clinical relevance.

For translational use, AI models should be evaluated according to whether they improve the selection of candidates that survive biological validation. A model is most useful when it identifies why a candidate is prioritized, what evidence supports the prediction, what uncertainty remains, and which experiment should be performed next. Therefore, AI-guided lncRNA discovery can be a prelude for validation planning, therapeutic feasibility assessment, and patient-stratification strategies.

### 5.1. AI Tasks in Translational lncRNA Discovery

AI can facilitate several steps in lncRNA target discovery if pertinent data is available, including transcript classification, coding-potential prediction, subcellular localization inference, interaction prediction, disease-association prediction, and candidate ranking [80]. These tasks are relevant because therapeutic strategy depends on whether the transcript is nuclear or cytoplasmic, whether it interacts with proteins or other RNAs, whether it is disease-context specific, and whether it can be modulated safely. Machine-learning models for lncRNA localization, for example, can help determine whether a candidate may be more suitable for antisense oligonucleotide, RNA interference, or transcriptional modulation strategies.

AI can also support functional prediction by integrating lncRNA–disease, lncRNA–protein, lncRNA–RNA, lncRNA–miRNA, and lncRNA–drug relationships [81]. Models for lncRNA–protein interaction prediction and RNA–RNA interaction prediction may help generate mechanistic hypotheses, especially when combined with expression, localization, and pathway evidence. However, these predictions should not be interpreted as proof of binding, mechanism, or therapeutic relevance.

For target prioritization, the most useful AI systems are those that combine disease relevance, genetic support, regulatory annotation, cell-type specificity, network context, therapeutic accessibility, and biomarker potential [9]. A transparent scoring approach may sometimes be more useful than a complex deep-learning model if it clearly identifies evidence gaps and guides follow-up validation. In this context, AI should help narrow the candidate list rather than replace experimental decision-making.

### 5.2. Modeling Strategies for Evidence Integration

Classical machine-learning models, including logistic regression, support vector machines, random forests, gradient boosting, and regularized regression, remain useful for curated lncRNA feature sets [82]. These approaches can integrate and quantify GWAS support, expression specificity, chromatin annotation, QTL evidence, localization, and disease association. They also provide important baselines for evaluating whether more complex models offer real improvement.

Deep-learning methods, including autoencoders, convolutional neural networks, recurrent neural networks, graph neural networks, attention-based models, and transformer architectures, can model nonlinear relationships across high-dimensional genomic and multi-omics datasets [83,84]. Applications in tumour genomics further demonstrate the potential of these architectures to integrate genomic, transcriptomic, epigenomic, and clinical data, while also highlighting persistent challenges related to interpretability, data scarcity, and external validation. Graph-based and transformer-based models are particularly relevant because lncRNAs are connected to variants, genes, proteins, pathways, drugs, tissues, and phenotypes [10]. Examples such as GCNFORMER [85] and iGATTLDA [86] illustrate how graph convolution and attention-based strategies have been applied to lncRNA–disease association prediction.

Knowledge graphs and heterogeneous information networks can be valuable for connecting lncRNAs to broader biomedical systems, but they also carry risks. A highly connected node may reflect genuine biology, but it may also reflect publication bias, database incompleteness, or repeated reuse of the same curated associations. Therefore, graph-based prioritization should include ablation testing, independent validation, and careful separation of training and test data [87]. The goal is not to rediscover well-known lncRNAs, but to identify non-obvious candidates with reproducible biological and translational evidence that might have been missed by manual-annotation approaches.

### 5.3. Explainability, Bias, and Validation of AI Predictions

Explainability is important because AI-ranked lncRNA candidates must be interpretable enough to guide experimental validation [88]. Feature attribution, pathway interpretation, graph ablation, counterfactual analysis, and Shapley Additive Explanations (SHAP)-style approaches can help identify which evidence layers contributed to a prediction. In genomics, explainable AI can reveal regulatory features or sequence patterns, but these outputs still require biological confirmation. A model explanation should therefore be treated as a guide for hypothesis testing, not as mechanistic proof.

Bias is a major concern in lncRNA prediction because annotation is incomplete, negative labels are often uncertain, and many datasets are biased toward well-studied diseases, tissues, populations, and evolutionarily conserved or well-characterized transcripts [11]. Additional sources of bias include tissue mismatch, ancestry imbalance, batch effects, class imbalance, publication bias, and data leakage. These issues are especially important for lncRNA–disease association models, where a poorly characterized but truly causative transcript may be incorrectly discarded as a false negative.

Validation should therefore extend beyond internal performance metrics such as accuracy, area under the receiver operating characteristic curve, or area under the precision–recall curve. For translational target discovery, stronger validation includes external cohort replication, calibration assessment, disease-specific testing, leakage checks, and prospective experimental follow-up. The most convincing AI-guided prediction would be a prioritized lncRNA that subsequently exhibits reproducible disease-context expression, successful experimental validation within the disease-phenotype context, mechanistic plausibility, and therapeutic feasibility. The main AI and machine-learning approaches that can be used for lncRNA target prioritization are summarized in Table 4, including their anticipated applications, input data, limitations, and representative references.

### 5.4. Evaluation and Reporting Standards for AI Prioritization

AI-based lncRNA prioritization should be evaluated according to its intended output. For binary or probabilistic predictions, discrimination should be reported using the area under the receiver operating characteristic curve and the area under the precision–recall curve, with the latter being particularly informative for imbalanced datasets [89]. When model outputs are intended to represent probabilities, calibration should be assessed using a calibration plot, calibration-in-the-large or intercept, and calibration slope, while overall probabilistic accuracy can be summarized using the Brier score [90].

For candidate-ranking systems, evaluation should include precision@K, recall@K, and the enrichment of independently supported or experimentally validated candidates among the top-ranked results. Generalizability should be assessed using entity- or context-held-out evaluation, such as holding out lncRNAs, diseases, cohorts, tissues, ancestry groups, or later time periods, depending on the intended application. Graph-based models should additionally prevent overlap or information leakage between training and test edges, clearly distinguish between transductive and inductive evaluation, and explicitly report the negative-sampling strategy because unobserved associations should not automatically be treated as confirmed negatives [91,92].

The most translationally informative endpoint is prospective validation yield: the proportion of top-ranked candidates that replicate in independent datasets and produce reproducible effects under orthogonal experimental validation.

## 6. Experimental and Translational Validation of Candidate lncRNA Targets

A candidate disease-causative lncRNA should not be described as a therapeutic target until its expression, function, mechanism, disease-model relevance, and therapeutic feasibility have been evaluated [93]. This is especially important because lncRNA associations are often derived from expression differences, genetic proximity, or computational prediction, none of which is sufficient to establish causality or druggability. Validation should therefore test whether the candidate transcript contributes to disease biology in a reproducible and therapeutically interpretable manner.

The validation strategy should be selected according to the presumed biology of the candidate. Some lncRNAs act through mechanisms dependent on the nuclear or cytoplasmic function and/or interactions of the mature RNA molecule, whereas others function through transcriptional activity, chromatin regulation, RNA structure, enhancer effects, or RNA–protein complexes [94]. These distinctions matter because ASO knockdown, siRNA, CRISPR interference, CRISPR deletion, Cas13 targeting, and overexpression do not perturb the same biological entity. A clear validation plan should therefore connect the disease hypothesis, perturbation strategy, mechanism, and intended therapeutic modality.

### 6.1. Expression and Disease-Context Validation

The first validation step is to confirm that the candidate lncRNA is expressed in the relevant disease tissue, cell type, or pathological state [55]. This should include confirmation of transcript identity, strand specificity, isoform structure, and disease association using independent RNA-seq datasets, qRT-PCR, targeted RNA capture, or long-read sequencing. For lncRNAs, transcript-level validation is particularly important because gene symbols may conceal multiple isoforms with different regulatory roles or therapeutic accessibility.

Bulk tissue analysis can be informative, but it may obscure cell-type-specific lncRNA expression. Single-cell and spatial assays are valuable when disease tissue contains mixed immune, stromal, epithelial, vascular, or neuronal populations [95]. These approaches can determine whether the candidate is expressed in the disease-relevant cellular compartment rather than in a secondary or contaminating cell population [96]. For example, single-cell analysis of the cardiac non-coding transcriptome has identified disease-associated lncRNA activity in specific cardiac cellular contexts.

Clinical validation should then assess whether lncRNA expression is associated with disease severity, prognosis, molecular subtype, therapeutic resistance, or treatment response [97]. However, clinical association should be interpreted carefully. A lncRNA may be useful as a biomarker without being a causal target. Therefore, expression validation should be presented as evidence of disease relevance, not as proof of therapeutic function.

### 6.2. Functional Perturbation and Mechanistic Validation

Functional validation should test whether altering the candidate lncRNA changes a disease-relevant phenotype. Depending on the candidate mechanism, perturbation may involve ASO-mediated knockdown, siRNA, CRISPR interference, CRISPR activation, CRISPR deletion, Cas13-mediated RNA targeting, overexpression, or rescue experiments [98]. RNA-targeting CRISPR systems such as Cas13 can support transcript-level perturbation without directly editing the DNA locus [99].

Mechanistic validation should define how the lncRNA influences disease biology [100]. Relevant assays may include RNA–protein interaction testing, chromatin occupancy analysis, RNA structure probing, enhancer activity assays, localization studies, transcriptomic profiling after perturbation, and pathway-level readouts. For example, lncRNA mechanisms may involve chromatin architecture, R-loop formation, RNA–protein scaffolding, transcript stabilization, or pathway regulation. A convincing mechanism should connect molecular interaction to downstream phenotype in the same disease-relevant system.

Rescue experiments are especially important because lncRNA perturbation can produce misleading effects through off-target oligonucleotide activity, guide-RNA artifacts, neighboring-gene disruption, or nonspecific stress responses [101]. A stronger target claim requires consistent results across complementary perturbation approaches and restoration of phenotype when the relevant transcript or pathway is rescued. For therapeutic translation, the most informative perturbation is usually the one that best reflects the intended clinical modality.

### 6.3. Translational Validation and Target Qualification

After expression and mechanistic evidence are established, candidate lncRNAs should be evaluated for translational feasibility. This includes target accessibility, direction of modulation, therapeutic modality, delivery route, target engagement, pharmacodynamic readouts, and preliminary safety considerations. A biologically important lncRNA may still be a weak therapeutic candidate if it is inaccessible, broadly required in normal tissues, difficult to measure, or unsuitable for safe modulation.

Model selection is also critical. Many lncRNAs show limited sequence conservation or strong context specificity, so standard animal models may not reproduce human lncRNA function [27]. Patient-derived organoids, iPSC-derived disease models, perturb-seq systems, xenografts, and primary cell models may help bridge this gap when they capture the relevant human disease state [102]. For candidates intended for RNA therapeutics, validation should also assess whether ASO, siRNA, CRISPR-based, RNA-editing, or small-molecule approaches can achieve meaningful modulation in the target tissue.

A practical validation hierarchy should comprise three sequential stages. The first establishes transcript certainty and disease-context expression. The second demonstrates functional perturbation, mechanism, and a disease-relevant phenotype. The third evaluates therapeutic modulation, delivery feasibility, safety, pharmacodynamic measurement, and patient-selection strategy.

## 7. RNA Therapeutic Strategies and Clinical Translation

RNA therapeutics provide a credible route for modulating disease-relevant lncRNAs, but therapeutic success depends on matching target biology with an appropriate modality, delivery system, pharmacodynamic readout, and safety profile. A candidate lncRNA may be biologically important, but it cannot be considered translationally mature unless it can be modulated in the relevant tissue and measured through reliable target-engagement or pathway-response assays. Therefore, lncRNA therapeutic development should begin early in the prioritization process rather than after target validation is complete.

The therapeutic strategy should be guided by the candidate’s subcellular localization, mechanism of action, disease context, direction of modulation, and tissue accessibility. Nuclear lncRNAs may be more suitable for antisense oligonucleotides, RNase H-dependent gapmers, splice-switching approaches, or transcriptional modulation, whereas cytoplasmic lncRNAs may be more compatible with siRNA or RNA interference strategies [103]. Other candidates may require CRISPR-based repression or activation, RNA editing, small-molecule targeting of RNA structures, or modulation of lncRNA-associated protein complexes [104].

### 7.1. Therapeutic Modalities for Targeting lncRNAs

Antisense oligonucleotides are among the most relevant modalities for lncRNA targeting because they can be designed to degrade, block, splice-modulate, or sterically inhibit specific RNA transcripts [105]. For nuclear lncRNAs, RNase H-dependent gapmers are particularly attractive because they can reduce target RNA abundance in the nucleus [106]. However, ASO development requires careful sequence selection, chemical modification, off-target screening, target-engagement assessment, and disease-relevant delivery planning [107]. Preclinical examples, such as systemic antisense targeting of NIS-lncRNA in neuropathic pain, illustrate how lncRNA modulation can produce disease-relevant effects in vivo when target biology and delivery are appropriately matched [67].

RNA interference approaches may be useful for cytoplasmic lncRNAs, especially when transcript knockdown is the intended therapeutic direction. The broader clinical progress of siRNA therapeutics supports the feasibility of RNA-based modulation, but success has been strongest for accessible tissues such as the liver and should not be generalized automatically to all lncRNA targets or disease sites [108]. For extrahepatic diseases, including cardiovascular, neurological, immune, and oncological indications, delivery remains a major determinant of translational feasibility.

CRISPR-based technologies, RNA-targeting Cas systems, RNA editing, and small molecules provide additional strategies for lncRNA modulation, but their therapeutic readiness varies. CRISPR interference and activation are powerful validation tools, yet clinical use requires stringent control of delivery, specificity, reversibility, and long-term effects. RNA-targeting systems such as Cas13 can support transcript-level perturbation and interaction mapping, while small molecules may be suitable for lncRNAs with defined structural motifs or disease-relevant RNA–protein interfaces [99]. For each modality, the key translational question is whether the intervention can modulate the correct molecular entity in the correct tissue at a clinically meaningful dose.

### 7.2. Delivery, Tissue Specificity, and Target Engagement

Delivery is one of the main barriers to lncRNA therapeutic translation. Potential delivery approaches include GalNAc conjugates, lipid nanoparticles, antibody–oligonucleotide conjugates, polymeric nanoparticles, viral vectors, exosome-based systems, and local administration routes [109]. GalNAc-conjugated siRNAs have created a strong precedent for liver-directed RNA therapeutics, but this success reflects favorable hepatocyte uptake biology and cannot be assumed for brain, heart, lung, tumor, or immune-cell targets [110].

Lipid nanoparticles and other nanocarriers can expand delivery options, but tissue selectivity, endosomal escape, immunogenicity, repeat dosing, biodistribution, and manufacturing consistency remain important challenges [111]. Brain delivery is particularly difficult because systemic nucleic acid therapeutics must overcome the blood–brain barrier. Emerging strategies such as peptide-functionalized lipid nanoparticles and blood–brain barrier-penetrating ASO systems may improve central nervous system access, but their applicability to lncRNA targets requires disease-specific validation [112].

Target engagement should be incorporated into therapeutic development from the beginning [113]. For lncRNA-targeting approaches, this may include measuring transcript knockdown, splice modulation, altered localization, pathway correction, protein-complex disruption, or downstream pharmacodynamic biomarkers. The most useful readouts are those that connect molecular modulation to disease-relevant biology. Without target-engagement and pharmacodynamic evidence, apparent therapeutic effects may be difficult to interpret and may not support clinical progression.

### 7.3. Safety, Clinical Translation, and Patient Selection

Safety evaluation is essential because RNA therapeutics can produce off-target hybridization, innate immune activation, hepatotoxicity, nephrotoxicity, altered transcriptome-wide regulation, or unintended effects in normal tissues [114]. For lncRNAs, safety assessment is complicated by tissue-specific function, incomplete annotation, overlapping transcription, and possible effects on neighboring genes. Therefore, candidate selection should include early review of normal tissue expression, conservation, essentiality, sequence specificity, and predicted off-target effects.

Clinical translation also requires a clear patient-selection strategy [115]. Some lncRNA-targeting approaches may be suitable only for patients with specific genotypes, molecular subtypes, transcript-expression patterns, resistance mechanisms, or pathway activation states. Companion diagnostics may therefore need to measure lncRNA expression, disease-associated variants, molecular subtype, or pharmacodynamic pathway markers. In this context, lncRNAs may function not only as therapeutic targets but also as biomarkers for stratification, response monitoring, or treatment resistance.

**Figure 3:**
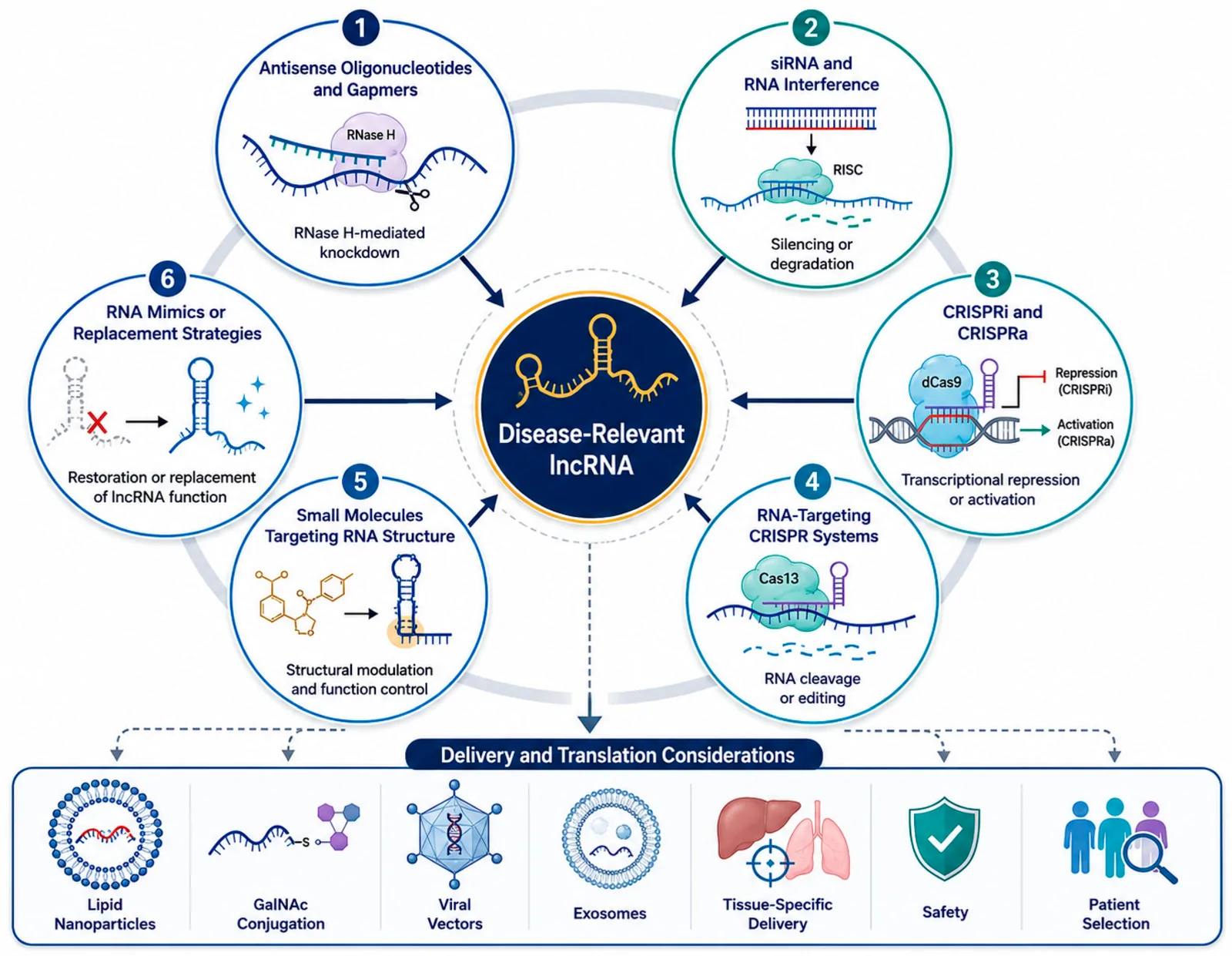
RNA therapeutic strategies for targeting disease-relevant lncRNAs. Candidate lncRNAs may be inhibited, restored, transcriptionally modulated, edited, structurally targeted, or used for patient stratification. Successful translation depends on matching the lncRNA mechanism with the appropriate therapeutic modality, delivery route, target-engagement assay, safety profile, and clinical endpoint.

The development pathway should therefore link target biology with modality choice, delivery feasibility, safety, pharmacodynamics, and clinical endpoints. A candidate lncRNA supported by genetic and multi-omics evidence may still fail translationally if it cannot be safely modulated in the relevant tissue or if patient benefit cannot be measured. Conversely, a candidate with modest discovery evidence but strong disease-context expression, validated perturbation response, accessible tissue distribution, and measurable pharmacodynamic markers may be more realistic for early therapeutic development. Figure 3 summarizes the principal RNA therapeutic strategies for modulating disease-relevant lncRNAs and highlights the need to align modality choice with delivery, target engagement, safety, and clinical implementation.

## 8. Challenges and Future Perspectives

Despite rapid progress in lncRNA biology, multi-omics profiling, and biomedical AI, AI-guided lncRNA target discovery remains limited by incomplete annotation, weak causal evidence, model bias, tissue-specific biology, and therapeutic delivery barriers [116]. These limitations are particularly important because a lncRNA may be statistically associated with disease but still lack validated function, mechanistic clarity, target accessibility, or safe therapeutic modulation. Future progress will depend on improving both the biological evidence base and the translational standards used to advance candidate lncRNAs.

A central challenge is evidence integration [117]. Genetic association, expression change, epigenomic activity, network position, and AI ranking each provide useful information, but none is sufficient alone. The strongest candidates will be those supported by convergent evidence across human genetics, disease-context expression, functional perturbation, mechanistic validation, therapeutic feasibility, and clinical relevance.

### 8.1. Biological and Annotation Challenges

lncRNA annotation remains incomplete and dynamic [118]. Long-read sequencing and updated reference resources continue to revise transcript boundaries, isoform structures, and gene models, which can affect candidate selection and experimental design. This is especially relevant for therapeutic development because an intervention designed against the wrong isoform, strand, or transcript boundary may fail to modulate the intended biological target.

Biological interpretation is also complicated by low expression, cell-type specificity, overlapping transcription, limited conservation, and context-dependent localization [119]. Some lncRNAs may act through the mature RNA molecule, whereas others may reflect transcriptional activity, enhancer function, chromatin architecture, or neighboring-gene regulation. These distinctions must be clarified before selecting a perturbation method or therapeutic modality.

Future studies should therefore report transcript identifiers, genome builds, isoforms, strand information, disease context, cellular source, and assay conditions whenever possible [30,66]. Negative or inconclusive findings should also be reported, and candidates should be downgraded when critical uncertainties remain. These include unresolved transcript or isoform identity, confounding effects involving neighbouring genes or enhancers, absence of disease-relevant expression, and poor concordance between human and model-system biology [27,55,66]. Additional concerns include RNA structural features that limit target accessibility, dependence on a single perturbation reagent, high normal-tissue exposure, predicted off-target hybridization, innate immune activation, and the absence of a realistic delivery route [14,33,101,114].

A candidate that fails one or more essential translational criteria may remain useful as a biomarker, disease-state reporter, or mechanistic probe, but it should not be presented as a qualified therapeutic target without additional evidence [6,13].

### 8.2. Computational and AI-Related Challenges

Dataset limitations also arise from inconsistent lncRNA identifiers and annotation releases, as well as from short-read measurements that may not resolve the transcript boundaries and isoforms relevant to therapeutic targeting [30,72]. Additional concerns include mismatch between healthy reference tissues and disease-relevant tissues or cell states, underrepresentation of diverse ancestry groups, and limited transferability of genetic and regulatory signals across populations [46,55,116]. lncRNA–disease prediction datasets are further constrained by sparse experimentally validated negative examples and repeated reuse of the same curated association databases across model development and evaluation, which may introduce label uncertainty, circularity, and data leakage [9,11,87]. Studies should therefore document the annotation release, genome build, transcript identifier, sample-inclusion criteria, missing-data handling, and any overlap among discovery, training, validation, and test resources [90].

AI models for lncRNA prioritization are limited by the quality of the datasets used to train and validate them [90]. Common problems include uncertain negative labels, class imbalance, tissue mismatch, ancestry imbalance, batch effects, incomplete annotation, publication bias, and data leakage. These issues are particularly important in lncRNA–disease association prediction because many unstudied lncRNAs may be incorrectly treated as true negatives.

Model performance should therefore be interpreted cautiously. High internal accuracy does not necessarily mean that a model can identify clinically meaningful therapeutic targets. External validation, leakage checks, calibration assessment, disease-specific testing, and prospective experimental follow-up are more informative than internal benchmarking alone. Explainable AI can help identify the evidence supporting a prediction, but model explanations should guide experiments rather than replace them.

Future AI systems should move beyond candidate ranking toward evidence-aware prioritization. Ideally, each model-ranked lncRNA should be accompanied by an explicit evidence profile showing genetic support, disease-context expression, regulatory evidence, predicted mechanism, therapeutic feasibility, uncertainty, and recommended validation experiments.

### 8.3. Translational Standards for lncRNA Target Discovery

A major future need is a clearer translational standard for distinguishing lncRNA biomarkers, disease regulators, and therapeutic targets. A biomarker candidate may require reproducible disease association and clinical validation, whereas a therapeutic target requires stronger evidence, including causal function, perturbation response, mechanism, target accessibility, safety plausibility, and therapeutic modulation [120].

Delivery and safety should also be considered earlier in target prioritization [121]. A lncRNA with strong biological relevance may still be unsuitable for therapeutic development if it is broadly expressed in essential normal tissues, inaccessible to current delivery systems, difficult to monitor pharmacodynamically, or likely to produce off-target effects. Conversely, candidates located in accessible tissues, measurable compartments, or genetically defined patient subgroups may be more feasible for early translational development.

Future lncRNA precision medicine will require closer integration of long-read transcriptomics, single-cell and spatial omics, multi-ancestry genetics, perturbation screening, RNA therapeutic chemistry, delivery engineering, and clinically annotated cohorts [122]. The goal should not be to expand candidate lists indefinitely, but to identify a smaller number of lncRNAs with sufficient evidence to justify therapeutic development.

## 9. Conclusions

lncRNAs represent a promising but still challenging class of therapeutic candidates in precision medicine. Their relevance arises from their roles in non-coding genomic regulation, tissue- and cell-type-specific expression, disease-associated molecular networks, and potential compatibility with RNA-based therapeutic strategies. However, disease association, differential expression, or computational prediction alone is insufficient to establish a lncRNA as a therapeutic target [66].

AI and machine learning can improve lncRNA target discovery by integrating GWAS, QTL, transcriptomic, epigenomic, single-cell, spatial, interaction, clinical, and therapeutic-feasibility data. Their main value is to prioritize candidates, reveal evidence gaps, and guide validation experiments. AI-guided ranking should therefore be interpreted as decision support rather than proof of causality, mechanism, druggability, or clinical utility.

The strongest path forward is a stepwise translational pipeline that connects non-coding genetic evidence, multi-omics convergence, disease-context expression, experimental perturbation, mechanistic validation, RNA therapeutic modality selection, delivery assessment, safety evaluation, and biomarker-informed patient stratification. Such a framework can help distinguish lncRNAs that are useful biomarkers or disease-state reporters from those with sufficient evidence to progress as therapeutic targets. By combining computational prioritization with rigorous biological and translational validation, lncRNA research can move toward more credible RNA-based precision medicine applications.

## Figures and Tables

**Table 1:** lncRNA classes and relevance to therapeutic target discovery.

lncRNA Class	Key Feature	Therapeutic Relevance	Main Limitation	Reference
Long intergenic ncRNA	Located between protein-coding genes	May act as independent regulatory targets	Often lowly expressed and incompletely annotated	[28]
Antisense lncRNA	Transcribed from the opposite strand of another gene	May regulate disease genes in a locus-specific manner	Indirect perturbation of sense-strand gene may complicate phenotypic readouts	[29]
Intronic lncRNA	Derived from intronic regions	May link intronic disease-associated variants to splicing or RNA regulation	Effects may be hard to separate from host-gene regulation	[30]
Bidirectional lncRNA	Transcribed near coding-gene promoters in the opposite direction	May reflect or regulate disease-associated promoter activity	Independence from nearby genes must be validated	[7]
Enhancer-associated lncRNA, eRNA	Transcribed from enhancer-like regions	May indicate disease-relevant regulatory elements	May be a marker rather than a functional driver	[31]
Pseudogene-derived lncRNA	Transcribed from pseudogene loci	May influence oncogenic, metabolic, or immune pathways	Sequence similarity can cause mapping and off-target issues	[32]

Note: These classes are not mutually exclusive. Therapeutic interpretation requires transcript-level annotation, disease-context expression, and functional validation.

**Table 2:** Evidence layers for prioritizing GWAS-derived lncRNA candidates.

Evidence Category	Evaluation Focus	Supporting Evidence	Translational Relevance	Reference
Genetic evidence	Link between the lncRNA locus and disease risk	GWAS signal, credible sets, fine-mapping, ancestry-specific associations	Provides the human genetic entry point for target discovery	[2]
Regulatory evidence	Variant effect on lncRNA regulation	eQTL, sQTL, chromatin accessibility, enhancer or promoter annotation, methylation data	Connects non-coding variants to possible lncRNA regulatory mechanisms	[59]
Disease-context evidence	lncRNA activity in relevant disease tissue or cell type	Bulk RNA-seq, single-cell RNA-seq, spatial transcriptomics, clinical cohort data	Supports disease relevance and guides model selection	[60]
Functional evidence	Effect of lncRNA perturbation on disease-relevant biology	Antisense oligonucleotide (ASO), siRNA, CRISPRi/a, Cas13, overexpression, rescue experiments	Distinguishes causal candidates from correlative biomarkers	[61]
Therapeutic feasibility	Practicality of safe and effective lncRNA modulation	Target accessibility, modality choice, delivery route, safety profile, pharmacodynamic readouts	Determines readiness for RNA therapeutic development	[62]

**Table 3:** Public resources for AI-guided lncRNA target discovery.

Resource	Primary Use	Key lncRNA Limitation	Recommended Safeguard	Reference
GENCODE, NONCODE, and LNCipedia	Gene, transcript, isoform, strand, and genomic-overlap annotation	Gene models and transcript identifiers differ across databases and annotation releases	Record the database release, genome build, transcript identifier, and strand	[5,19,21]
RNAcentral and lncATLAS	Sequence retrieval and subcellular-localization assessment	Sequence or localization alone does not establish disease relevance or causality	Use these resources for annotation and therapeutic-modality assessment rather than as causal evidence	[22,23]
GTEx and GTEx eQTL resources	Healthy-tissue expression and genetic-regulatory effects	Healthy adult tissues may not represent disease-associated tissues or cell states	Integrate with disease-tissue, single-cell, spatial, and functional evidence	[59]
TCGA and GDC	Cancer expression, molecular subtype, clinical outcome, and multi-omics analysis	Bulk-tumour composition, short-read quantification, and treatment-related confounding may affect lncRNA interpretation	Validate findings in independent cohorts and transcript-resolved assays	[69]
ENCODE	Chromatin accessibility, promoter, enhancer, and regulatory annotation	Regulatory profiles depend on the cell type, experimental system, and biological context	Match the regulatory assay and cellular context to the disease hypothesis	[70]
GEO	Independent expression, perturbation, and functional-validation datasets	Heterogeneous protocols, variable data quality, and incomplete metadata	Apply consistent reprocessing and report dataset-selection and inclusion criteria	[71]

Note: These resources provide complementary evidence rather than interchangeable measurements. Integrative analyses should therefore preserve the provenance and uncertainty of each data layer and should avoid treating database membership, sequence annotation, or differential expression as independent evidence of causality.

**Table 4:** Representative AI and machine-learning approaches for lncRNA target prioritization.

Approach and Representative Model	Application	Inputs	Strength	Limitation	Reference
Interpretable machine learning: regularized regression, random forest	Transparent candidate ranking	GWAS, QTL, expression, chromatin, and annotation features	Relatively interpretable and suitable for curated datasets	May miss complex nonlinear relationships	[82]
Multimodal deep learning: autoencoders, neural networks	Integration of high-dimensional multi-omics data	RNA sequence, transcriptomic, epigenomic, proteomic, and clinical data	Learns shared patterns across heterogeneous data layers	Requires large, harmonized datasets and may be difficult to interpret	[83]
Heterogeneous graph models: GCNFORMER, iGAT-TLDA	Prediction of lncRNA–disease and molecular-network associations	lncRNAs, genes, proteins, pathways, diseases, drugs, and interactions	Represents relationships among multiple biological entities	Sensitive to incomplete networks, literature bias, and data leakage	[85,86]
Transformer and foundation models	Sequence and regulatory representation learning	DNA/RNA sequences, regulatory genomics, and single-cell profiles	Captures long-range sequence and regulatory dependencies	Can inherit annotation, tissue, and population biases	[83]
Causal and probabilistic models	Direction-aware prioritization and uncertainty estimation	GWAS, QTL, perturbation, longitudinal, and multi-omics data	Helps distinguish predictive association from evidence compatible with causality	Depends on assumptions that may not hold in observational data	[54]
Explainable AI: feature attribution, SHAP, graph ablation	Interpretation of ranked candidates and evidence gaps	Model features, pathway scores, network structure, and predictions	Identifies evidence contributing to candidate prioritization	Explanations may reflect confounding or model artefacts	[65,88]

## Data Availability

No new data were created or analyzed in this study. Data sharing is not applicable to this article.
